# *Lactobacillus plantarum* TWK10 Supplementation Improves Exercise Performance and Increases Muscle Mass in Mice

**DOI:** 10.3390/nu8040205

**Published:** 2016-04-07

**Authors:** Yi-Ming Chen, Li Wei, Yen-Shuo Chiu, Yi-Ju Hsu, Tsung-Yu Tsai, Ming-Fu Wang, Chi-Chang Huang

**Affiliations:** 1 Graduate Institute of Sports Science, National Taiwan Sport University, Taoyuan 33301, Taiwan; 1021302@ntsu.edu.tw (Y.-M.C.); 1021301@ntsu.edu.tw (Y.-S.C.); 1041302@ntsu.edu.tw (Y.-J.H.); 2 Department of Neurosurgery, Taipei Medical University-WanFang Hospital, Taipei City 11696, Taiwan; nsweili@gmail.com; 3 Department of Orthopedic Surgery, Taipei Medical University-Shuang Ho Hospital, New Taipei City 23561, Taiwan; 4 School of Nutrition and Health Sciences, Taipei Medical University, Taipei 11031, Taiwan; 5 Department of Food Science, Fu Jen Catholic University, Taipei 24205, Taiwan; 6 Department of Food and Nutrition, Providence University, Taichung 43301, Taiwan

**Keywords:** *Lactobacillus plantarum* TWK10, exercise performance, forelimb grip strength, gastrocnemius muscles

## Abstract

*Lactobacillus plantarum* (*L. plantarum*) is a well-known probiotic among the ingested-microorganism probiotics (*i.e.*, ingested microorganisms associated with beneficial effects for the host). However, few studies have examined the effects of *L. plantarum* TWK10 (LP10) supplementation on exercise performance, physical fatigue, and gut microbial profile. Male Institute of Cancer Research (ICR) strain mice were divided into three groups (*n* = 8 per group) for oral administration of LP10 for six weeks at 0, 2.05 × 10^8^, or 1.03 × 10^9^ colony-forming units/kg/day, designated the vehicle, LP10-1X and LP10-5X groups, respectively. LP10 significantly decreased final body weight and increased relative muscle weight (%). LP10 supplementation dose-dependently increased grip strength (*p* < 0.0001) and endurance swimming time (*p* < 0.001) and decreased levels of serum lactate (*p* < 0.0001), ammonia (*p* < 0.0001), creatine kinase (*p* = 0.0118), and glucose (*p* = 0.0151) after acute exercise challenge. The number of type I fibers (slow muscle) in gastrocnemius muscle significantly increased with LP10 treatment. In addition, serum levels of albumin, blood urea nitrogen, creatinine, and triacylglycerol significantly decreased with LP10 treatment. Long-term supplementation with LP10 may increase muscle mass, enhance energy harvesting, and have health-promotion, performance-improvement, and anti-fatigue effects.

## 1. Introduction

The species *Lactobacillus plantarum* (*L. plantarum*) is a gram-positive bacterium. Numerous investigations have revealed that *Lactobacillus* spp. has various biological effects, such as improving insulin sensitivity, the underlying cause of obesity-associated metabolic abnormalities [1]; inducing cancer cell apoptosis [2]; and antihypertensive [3], cholesterol-lowering [4], anti-inflammation [5], antimicrobial [6], and antioxidation activities [7]. These findings have stimulated an explosion of investigations of *Lactobacillus* spp., its bioactivities, and its possible role in human health.

Fatigue is defined as physiological fatigue, such as extreme fatigue after exercise, accompanied by poor athletic performance and loss of favorable working conditions for the organs [8]. However, fatigue is difficult to define because of the unique intrinsic properties and anatomic features of individual muscles [9]. Exercise leads to changes in metabolism; energy provision; and cardiovascular, respiratory, thermoregulatory, and hormonal responses [10]. During explosive sprinting, the energy supplied to rephosphorylate adenosine diphosphate (ADP) to adenosine triphosphate (ATP) is determined largely by the amount of phosphocreatine stored in the muscle [11]. During the gut fermentation process, probiotic bacteria can improve the energy harvesting capacity via production of short chain fatty acids (SCFAs) [12], and a recent study has revealed a tight and coordinated connection between gut microbes and host metabolism, energy utilization, and storage [13]. The main reason why *Lactobacillus* spp. may affect exercise performance is that *Lactobacillus* bacteria produce lactic acid, which could facilitate the production of butyrate by lactate-utilizing bacteria that first producing acetyl-CoA from lactate [14]. In the so-called classical pathway, the enzymes phosphotransbutyrylase and butyrate kinase convert butyryl-CoA to butyrate and coenzyme A with the concomitant formation of ATP. In addition, the gut microbiota has been proposed to influence muscle metabolism [15] and may constitute a future therapeutic target in the management of muscle wasting. Thus, the probiotic and gut microbiota could play important roles in maintaining normal physiology and energy production during exercise.

Antioxidant supplementation during high-intensity exercise can prolong exercise performance, reduce metabolite production, and reduce physical fatigue [16,17]; during low-intensity exercise, it may inhibit several steps in signal transduction and reduce exercise performance. This may occur because reactive oxygen species (ROS) act as mediators of normal and pathological signal transduction pathways [18]. Many studies have shown that *L. plantarum* has significant antioxidant activity both *in vitro* and *in vivo* [19,20]. However, few studies have focused on (probiotic) lactobacilli and their interaction with gut microbiota, and the possible ergogenic anti-fatigue function.

In this study, we evaluated the effects of *L. plantarum* TWK10 (LP10) supplementation on exercise performance, fatigue-associated biochemical indices, and gastrocnemius muscle fiber profile in mice [21]. We further examined a possible mechanism and gut microbiota to explore the mechanism of the probiotic effect on anti-fatigue activity.

## 2. Experimental Section

### 2.1. Materials, Animals, and Experiment Design

*L. plantarum* TWK10 (LP10) was obtained from Dr. Tsung-Yu Tsai (Department of Food Science, Fu Jen Catholic University, Taipei City, Taiwan). *L. plantarum* TWK10 was isolated from Taiwanese fermented cabbage and stored at −80 °C in *Lactobacilli* MRS (Difco Corp., Sparks, MD, USA) with 20% glycerol [22]. Before testing, the bacterial strains were retrieved from frozen storage and cultured in MRS broth for 24 h at 37 °C. Before supplementation, cells were centrifuged at 3000× *g* for 10 min and washed twice with phosphate buffered saline (PBS). Pellets were resuspended in PBS, pH 7.2. Male ICR mice (6 weeks old) grown under specific pathogen-free conditions were purchased from BioLASCO (Yi-Lan, Taiwan). All mice were provided a standard laboratory diet (No. 5001; PMI Nutrition International, Brentwood, MO, USA) and distilled water *ad libitum* and housed at 12-h light/12-h dark cycle at room temperature (22 °C ± 1 °C) and 50%–60% humidity. The Institutional Animal Care and Use Committee (IACUC) of National Taiwan Sport University (NTSU) inspected all animal experiments, and this study conformed to the guidelines of protocol IACUC-10405 approved by the IACUC ethics committee.

The human dose of LP10, 1 × 10^10^ CFU per day, was modified from previous studies [23,24]. The mouse LP10 dose (2.05 × 10^8^ CFU/kg) we used was converted from a human equivalent dose (HED) based on body surface area by the following formula from the US Food and Drug Administration: assuming a human weight of 60 kg, the HED for 1 × 10^10^ colony-forming units (CFU) ÷ 60 (kg) = 16.67 × 10^7^ × 12.3 = a mouse dose of 2.05 × 10^8^ CFU/kg; the conversion coefficient 12.3 was used to account for differences in body surface area between a mouse and a human, as previously described [25].

In total, 24 mice were randomly assigned to 3 groups (8 mice/group) for daily oral LP10 treatment for 6 weeks: vehicle, 2.05 × 10^8^ CFU/kg (LP10-1X), and 1.03 × 10^9^ CFU/kg (LP10-5X). The vehicle group received the same volume of solution equivalent to individual body weight (BW). Mice were randomly housed in groups of 4 per cage.

### 2.2. Forelimb Grip Strength Test

A low-force testing system (Model-RX-5, Aikoh Engineering, Nagoya, Japan) was used to measure the forelimb grip strength of treated mice as previously described [26].

### 2.3. Swimming Exercise Performance Test

The swim-to-exhaustion test involved loads corresponding to 5% of the mouse BW attached to the tail to evaluate endurance time as previously described [27]. The swimming endurance time of each mouse was recorded from beginning to exhaustion, determined by observing loss of coordinated movements and failure to return to the surface within 7 s.

### 2.4. Determination of Fatigue-Associated Biochemical Variables

The effect of LP10 supplementation on fatigue-associated biochemical indices was evaluated after exercise as previously described [28,29]. At 1 h after LP10 supplementation, all mice underwent a 15-min swim test without weight loading. After a 15-min swim exercise, blood samples were immediately collected and centrifuged at 1500× *g* and 4 °C for 10 min for serum separation. Serum lactate, ammonia, glucose, creatine kinase (CK) and blood urea nitrogen (BUN) levels were determined using the Beckman DxC 800 autoanalyzer (Beckman Coulter, Brea, CA, USA).

### 2.5. Clinical Biochemical Profiles

At the end of the experimental period, all mice were euthanized with 95% CO_2_ asphyxiation, and blood was immediately collected. Serum was separated by centrifugation, and levels of the clinical biochemical variables including CK, albumin, total protein (TP), BUN, creatinine, uric acid (UA), total cholesterol (TC), triacylglycerols (TG) and glucose were measured using the Beckman DxC 800 autoanalyzer.

### 2.6. Histology of Tissues

All tissues were carefully removed, minced, and fixed in 10% formalin. Samples were embedded in paraffin and cut into 4-μm thick slices for morphological and pathological evaluations. Tissue was stained with hematoxylin and eosin (H & E) and examined under a light microscope equipped with a CCD camera (BX-51, Olympus, Tokyo, Japan) by a veterinary pathologist.

### 2.7. Immunohistochemical Staining of Gastrocnemius Muscles

Target organs were carefully removed, minced, and fixed in 10% formalin after sacrifice. Tissues were embedded in paraffin and cut into 4-μm thick slices for morphological and pathological evaluations. Immunohistochemical (IHC) staining of tissues involved use of the Leica antibody to myosin heavy chain fast (WB-MHCf) and myosin heavy chain slow (WB-MHCs). By using automated BondMax with double staining, WB-MHCf and WB-MHCs epitope retrieval involved use of ER2 (AR9640) (pH 9) retrieval solution for 30 min once, followed by incubation with WB-MHCf and WB-MHCs antibodies at a 100-fold dilution for 30 min. The detection kit used was the Bond Polymer Refine Detection (DS9800) (incubation with post primary for 8 min, polymer for 8 min and 3′3′-diaminobenzidine for 5 min) and Bond Polymer Refine Red Detection (DS9390) (incubation with post primary for 20 min, polymer for 30 min, Red for 10 min and haematoxylin for 5 min). Finally, results were examined under a light microscope equipped with a CCD camera (BX-51, Olympus, Tokyo) by a veterinary pathologist.

### 2.8. Statistical Analysis

All data are expressed as mean ± SEM, *n* = 8 mice/group. Statistical differences among groups were analyzed by a one-way analysis of variance (ANOVA) and the Cochran-Armitage test for the dose-effect trend analysis with SAS 9.0 (SAS Inst., Cary, NC, USA). *p* < 0.05 was considered statistically significant. Differences between groups were analyzed by one-way ANOVA using Duncan’s *post-hoc* test, and *p* values < 0.05 were considered significant.

## 3. Results and Discussion

### 3.1. Effects of LP10 on Forelimb Grip Strength

**Figure 1:**
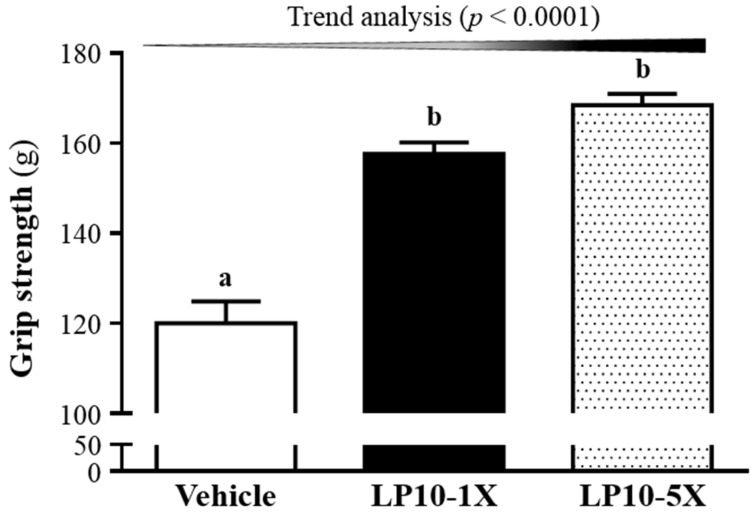
Effects of LP10 (*Lactobacillus plantarum* TWK10) supplementation for six weeks on forelimb grip strength. Mice were pretreated with vehicle, LP10-1X, or LP10-5X for six weeks, and then forelimb grip strength was tested. Data are mean ± SEM, 8 mice/group, by one-way ANOVA. Different letters (a, b) indicate a significant difference at *p* < 0.05.

After supplementation for six weeks, the forelimb grip strengths in the vehicle, LP10-1X, and LP10-5X groups were 120 ± 5, 158 ± 3, and 168 ± 3 g, respectively (Figure 1). Forelimb grip strengths were 1.31 and 1.40 fold higher in the LP10-1X and LP10-5X groups than in the vehicle treatment group (both *p* < 0.0001). In the trend analysis, absolute forelimb grip strength dose-dependently increased with increasing LP10 dose (*p* < 0.0001). In general, programmed exercise training is required to increase grip strength [27]; however, we found that LP10 supplementation benefited grip strength even though test animals did not undergo a training intervention. Thus, long-term LP10 supplementation could benefit the muscle explosive force when no training protocol is implemented, but few studies have investigated probiotics supplementation to improve muscle strength. In previous research, supplementation of *Lactobacillus* spp. reduced the expression of atrophy markers [30] and the levels of systemic inflammatory cytokines, and restoration of these *Lactobasilli* levels reduced the markers of the autophagy-lysosomal pathway, a major system of protein breakdown in gastrocnemius and tibialis muscle [31]. Thus, supplementation of LP10 in our present data increased muscle mass to enhance forelimb grip strength. On the other hand, supplementation of prebiotics or probiotics increased gut SCFA content. SCFAs produced by the microbiota in the cecum and the colon can be found in hepatic, portal, and peripheral blood [32,33]. These SCFAs affect lipid, glucose, and cholesterol metabolism in various tissues and the maintenance of gut integrity [34]. These might be possible mechanisms underlying the increase in muscle mass and strength.

### 3.2. Effect of LP10 on Exercise Performance in a Weight-Loaded Swimming Test

**Figure 2:**
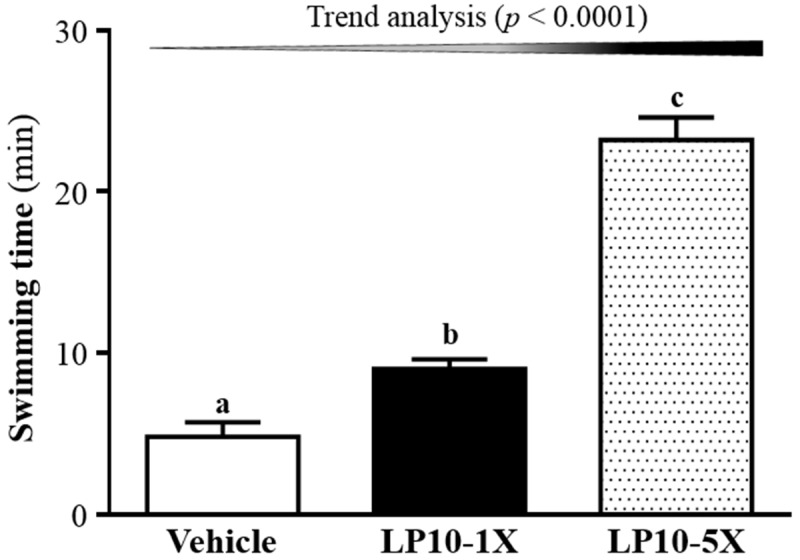
Effects of LP10 supplementation on swimming exercise performance. Mice were pretreated with vehicle, LP10-1X, LP10-5X for six weeks, and then performed an exhaustive swimming exercise with a load equivalent to 5% of body weight attached to the tail. Data are mean ± SEM, *n* = 8 mice/group, by one-way ANOVA. Different letters (a, b) indicate a significant difference at *p* < 0.05.

Energy metabolism during muscular activity determines the level of physiological fatigue [35]. An important index in evaluating anti-fatigue treatment is exercise endurance. Endurance swimming times were 4.8 ± 0.9, 9.0 ± 0.6, and 23.2 ± 1.4 min with vehicle, LP10-1X, and LP10-5X treatment, respectively (Figure 2). The exhaustive swimming time was longer, by 1.85 (*p* = 0.0183) and 4.81 folds (*p* < 0.0001), with LP-1X and LP-5X, respectively, than with vehicle treatment. In the trend analysis, endurance swimming time dose-dependently increased with increasing LP10 dose (*p* < 0.0001). Probiotics have a wide range of benefits to promote endurance exercise performance [36]. LP10 may improve endurance performance in the absence of training. Further investigation is required to elucidate the effects of LP10 supplementation combined with diverse gut microbiota and exercise training on endurance performance. In a recent study, certain probiotics (e.g., *Bacillus coagulans*) were shown to increase nutrient absorption, specifically protein absorption, in the form of better leucine absorption from whey protein [37]. Improved protein utilization could increase muscle mass and thereby enhance exercise performance.

### 3.3. Effect of LP10 Supplementation on Serum Lactate, Ammonia, Glucose, CK and BUN Levels after Acute Exercise Challenge

**Figure 3:**
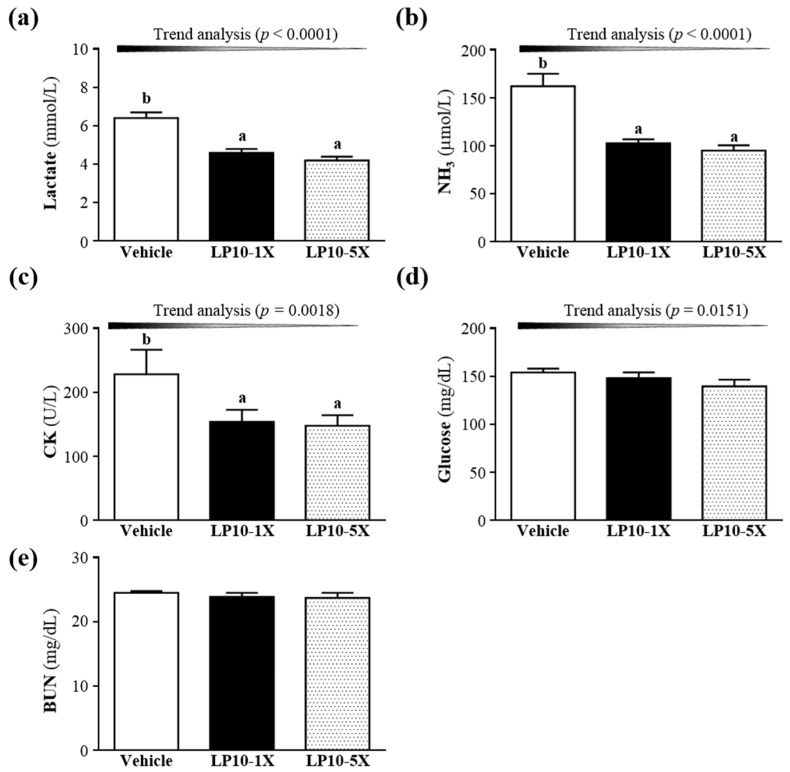
Effect of LP10 supplementation on serum levels of lactate (**A**); ammonia (**B**); creatine kinase (CK) (**C**); glucose (**D**); and blood urea nitrogen (BUN) (**E**) after acute exercise challenge. Data are mean ± SEM, *n* = 8 mice/group, by one-way ANOVA. Different letters (a, b) indicate a significant difference at *p* < 0.05.

Exercise-induced muscle fatigue can be evaluated by biochemical indicators such as lactate, ammonia, glucose, CK, and BUN levels [38,39]. Lactate accumulates in the blood and in the muscles engaged in the exercise and exceeds the aerobic metabolic capacity. When the lactic acid concentration increases, hydrogen ions accumulate, which leads to fatigue due to acidification [40,41]. Lactate levels in the vehicle, LP10-1X, and LP-5X groups were 6.4 ± 0.3, 4.6 ± 0.2, and 4.2 ± 0.7 mmol/L, with lower lactate levels with LP10-1X and LP10-5X supplementation (27.88%, *p* = 0.0004 and 34.11%, *p* = 0.0005, respectively) than with vehicle treatment (Figure 3A). In the trend analysis, serum lactate level was dose-dependently decreased with increasing LP10 dose (*p* < 0.0001). After acute exercise, relaxation is significantly affected by the blood lactate clearance rate. Approximately 75% of the total amount of lactate produced is used for oxidative production of energy in the exercising body, and it could be utilized for the *de novo* synthesis of glucose in the liver [42]. Clinical studies have found a positive correlation between probiotics supplementation and serum biochemical indicators [43,44]. Probiotics supplementation has rarely been used to investigate serum biochemical levels after acute exercise. LP10 supplementation may have potential for the removal and utilization of blood lactate after exercise.

Ammonia, another important metabolite produced during energy metabolism for exercise, is generated by different sources. Accumulation of ammonia in the blood and brain during exercise can negatively affect the central nervous system and cause fatigue. Although exercise-induced ammonia toxicity is transient and reversible depending on the disease state, it may affect continuing coordinated activity in critical regions of the central nervous system [45]. The central nervous system plays a crucial role in the development of physical fatigue. In a recent study, gastrointestinal inflammation induced anxiety-like behavior and altered the biochemistry of the central nervous system [46]. Thus, probiotics supplementation may play an important role in the central nervous system and fatigue. Serum ammonia levels were 162.1 ± 13, 102.5 ± 4.3, and 95 ± 5.5 μmol/L in the vehicle, LP10-1X, and LP10-5X groups, respectively (Figure 3B), and levels were lower, by 36.78% (*p* = 0.0004) and 41.40% (*p* = 0.0001), with LP10-1X and LP10-5X, respectively, than with vehicle treatment. Thus, continuous supplementation with LP10 for six weeks could decrease ammonia levels during exercise. Trend analysis showed that the serum ammonia level dose-dependently decreased with increasing LP10 dose (*p* < 0.0001).

The blood glucose level is an important index for performance maintenance during exercise [47]. The serum glucose levels were 154.1 ± 4, 148.3 ± 6, and 139 ± 7 mg/dL in the vehicle, LP10-1X, and LP10-5X groups, respectively, with no difference among groups (Figure 3C). Trend analysis showed that serum glucose levels dose-dependently decreased with increasing LP10 dose (*p* = 0.0151). Therefore, continuous supplementation with LP10 for six weeks could increase energy utilization and improve exercise performance.

Previously, we investigated the association of intestinal bacteria and exercise performance in mice. Gut microbial status could be crucial for exercise performance. Endurance swimming time was correlated with abundant gut microbiota in mice; in fact, the greater the amount of gut microbiota, the longer the endurance swimming time and the lower the serum glucose [48]. Therefore, gut microbiota can regulate different types of energy utilization in the host.

Serum CK level is an important clinical biomarker of muscle damage, muscular dystrophy, severe muscle breakdown, myocardial infarction, autoimmune myositides, and acute renal failure. High-intensity exercise challenge can physically or chemically cause tissue damage and muscular cell necrosis [49]. The serum CK concentration is reduced in the normal state but increased in muscle tissue with hypoxia and the accumulation of metabolites during exercise caused by muscle cell damage, which results in decreased exercise performance [50]. We found serum CK levels of 228.3 ± 38.4, 154.1 ± 18.8, and 147.8 ± 16.5 mg/dL in the vehicle, LP10-1X, and LP10-5X groups, respectively (Figure 3D), with lower levels with LP-1X and LP-5X, by 32.48% (*p* = 0.0165) and 35.27% (*p* = 0.0142), respectively, than with vehicle treatment. Therefore, LP10 supplementation could ameliorate skeletal muscle injury induced by acute exercise challenge. Trend analysis revealed that LP10 treatment had a significant dose-dependent effect on CK level (*p* = 0.0118).

BUN is an important biochemical parameter related to fatigue. The BUN level is used to measure the amount of nitrogen in blood from the waste product of urea. Urea serves an important role in the metabolism of nitrogen-containing compounds. Consequently, an increased BUN level reflects the decomposition of protein, which will adversely affect the contractive strength of muscle and lead to fatigue [27,28,51]. Serum BUN level did not differ among treatment groups (Figure 3E). Above all, LP10 may have potential as an ergogenic supplement by improving gut microbiota and regulating energy utilization.

### 3.4. General Characteristics of Mice with LP10 Supplementation for Six Weeks

**Figure 4:**
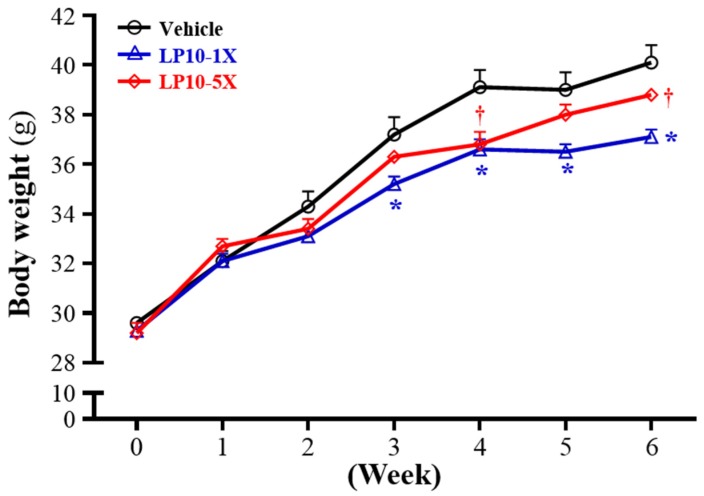
Change in body weight (BW) during the experiment. Data are mean ± SEM, *n* = 8/group. * *p* < 0.05, **^†^** *p* < 0.05 for LP10-1X and LP10-5X, respectively, compared with vehicle by one-way ANOVA.

Initial BW did not differ among the vehicle, LP10-1X, and LP10-5X groups (Table 1). After six-week supplementation with LP10, the final BW was lower with LP10-1X and LP10-5X, by 7.47% (*p* = 0.0003) and 3.46% (*p* = 0.0567), respectively, than with vehicle treatment. In addition, daily intake of diet and water increased in LP10-5X fed mice. Trend analysis showed that daily intake of diet (*p* < 0.0001) and water (*p* < 0.0001) dose-dependently increased with LP10 supplementation, so daily diet intake was increased but BW was decreased. In addition, BW was significantly lower (*p* < 0.05) with vehicle treatment at Week 3 of LP10 supplementation (Figure 4). Thus, three-week LP10 supplementation may change the body composition and energy utilization. Probiotic supplementation could reflect the biochemistry of the conversion of carbohydrates into short-chain fatty acids (SCFAs) by the change in the bacteria composition of the gut microbial community [52].

We measured the effect of LP10 on the muscle and epididymal fat pad (EFP) mass and relative tissue weight (different tissue weights adjusted for individual BW %). The EFP weight was lower by 34.62% (*p* = 0.003) and 50.30% (*p* < 0.0001) with LP10-1X and LP10-5X, respectively, than with vehicle treatment. Trend analysis showed that EFP weight dose-dependently decreased with LP10 supplementation (*p <* 0.0001). The relative weight of EFP (%) was lower by 28.93% (*p* = 0.0048) and 48.22% (*p* < 0.0001) with LP10-1X and LP10-5X, respectively, than with vehicle treatment. The relative weight (%) of muscle (gastrocnemius and soleus muscles) was greater by 1.10 (*p* = 0.0003) and 1.07 folds (*p* = 0.0098) with LP10-1X and LP10-5X, respectively, than with vehicle treatment. Trend analysis also showed a significant dose-dependent decrease and increase in relative EFP weight (%) and relative muscle weight (%), respectively, with LP10 supplementation. Thus, supplementation of LP10 for six weeks could change body composition to more fit and stronger. In addition, trend analysis showed significant increases in relative weight (%) of the kidney (*p* < 0.0001) and heart (*p* = 0.0018) with increasing LP10 dose. We found no gross abnormalities attributed to LP10 when weighing organs.

### 3.5. Effect of LP10 Supplementation on Biochemical Variables at the End of the Experiment

We observed beneficial effects of LP10 on grip strength and exhaustive exercise challenge, as well as other physiological effects, with six-week LP10 supplementation. We further investigated whether six-week LP10 treatment affected other biochemical markers in healthy mice. We examined tissue- and health status-related biochemical variables and major organs including skeletal muscle, heart, kidney, and lung (Table 2).

**Figure 5:**
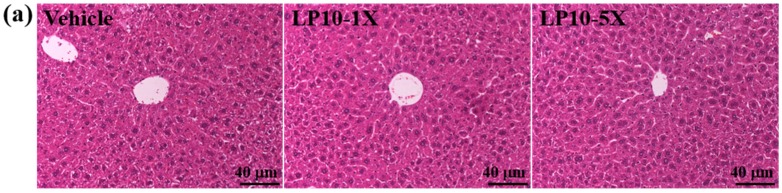

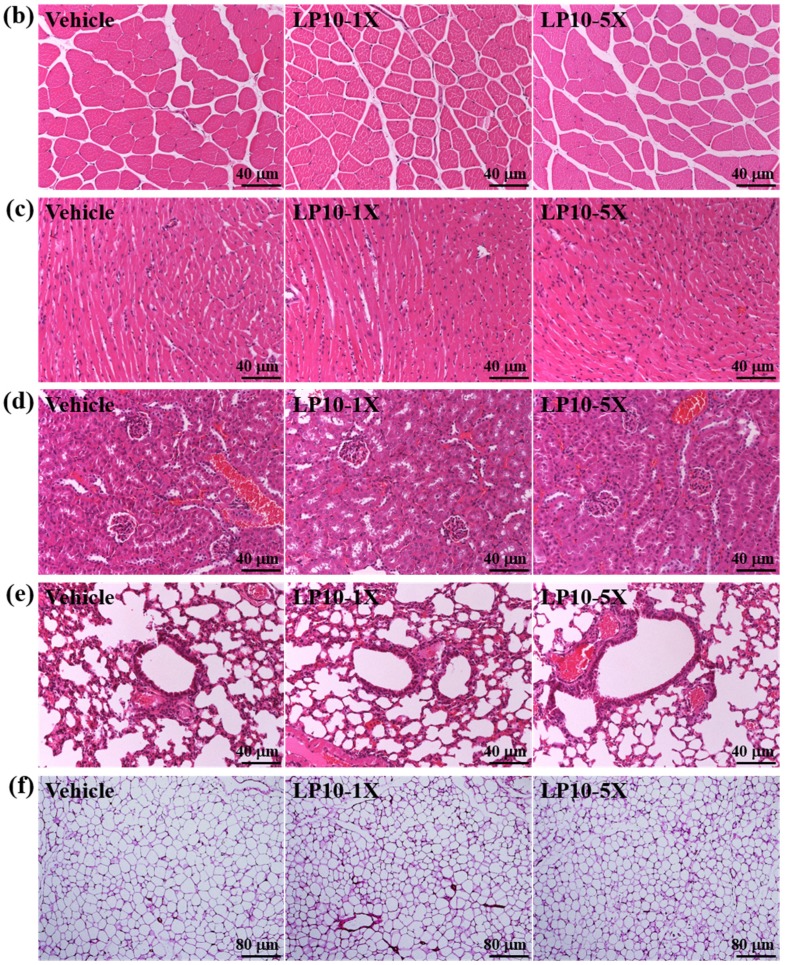
Effect of LP10 supplementation on morphology of: liver (**a**); skeletal muscle (**b**); heart (**c**); kidney (**d**); lungs (**e**); and epididymal fat pad (**f**). Specimens were photographed by light microscopy. (H & E stain, magnification: ×200). EFP: epididymal fat pad.

Levels of biochemical indices, including CK, TP, creatinine, UA, TC, and glucose, did not differ among groups (*p* > 0.05, Table 2). Serum albumin levels were lower by 7.64% (*p* *=* 0.0375) with LP10-5X than with vehicle treatment. Serum BUN levels were lower by 15.50% (*p* *=* 0.0218) and 13.29% (*p* = 0.0037) with LP10-1X and LP10-5X, respectively, than with vehicle treatment. On trend analysis, serum albumin (*p* = 0.0012) and BUN (*p* = 0.0017) levels were dose-dependently decreased with LP10 supplementation. Therefore, long-term daily supplementation with LP10 may have potential for tissue protection and renal benefits. In addition, serum level of TC, an index of lipid profile, was lower by 22.76% (*p* *=* 0.0069) and 26.60% (*p* = 0.0021) with LP10-1X and LP10-5X, respectively, than with vehicle treatment. Trend analysis showed significantly decreased serum TG levels (*p* = 0.0005) with increasing LP10 dose. According to a previous study, the beneficial lipid profile in *Lactobacillus* is attributed to the accumulation of SCFAs during fermentation [53]. SCFAs can transport from the intestinal lumen into the blood compartment of hosts and regulate the balance in fatty acid synthesis [54]. In addition, LP10 supplementation for six weeks had no adverse effects on major organs such as the liver, skeletal muscle, heart, kidney, lung, and EFP. Therefore, the dose of LP10 supplementation used in this study was safe (Figure 5).

### 3.6. IHC of Gastrocnemius Muscles for Type I and Type II Muscle Fibers

**Figure 6:**
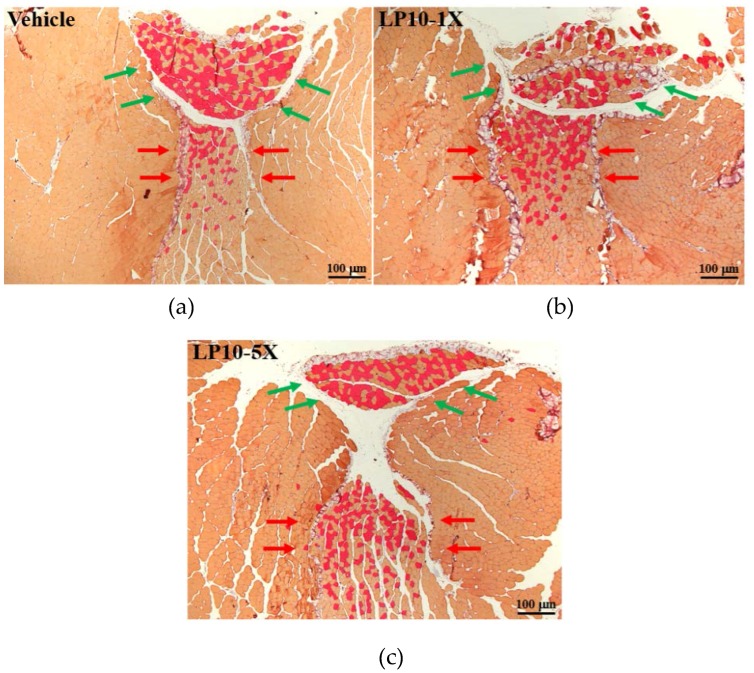
Immunohistochemistry (IHC) of effect of LP10 supplementation with: vehicle (**a**); LP10-1X (**b**); and LP10-5X (**c**) on type I and type II muscle fibers in gastrocnemius muscle. Red fibers are type I fibers; orange fibers are type II fibers. Specimens were photographed by light microscopy. (IHC stain, magnification: ×50).

**Figure 7:**
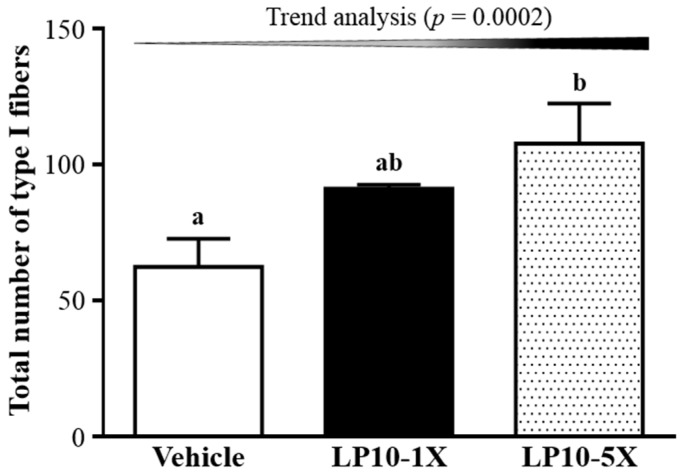
Total number of type I muscle fibers with LP10 supplementation in gastrocnemius muscle of mice. Data are mean ± SEM, *n* = 8 mice/group. Different letters (a, b) indicate a significant difference at *p* < 0.05 by one-way ANOVA.

IHC showed red fibers (slow muscle) as type I fibers and orange fibers (fast muscle) as type II fibers in gastrocnemius muscle of treated mice (Figure 6). Type I and II muscle fibers did not differ among treatments in soleus muscle but did differ in gastrocnemius muscle. The numbers of type I muscle fibers were 62 ± 10, 91 ± 2, and 108 ± 15 with vehicle, LP10-1X, and LP10-5X treatment, respectively (Figure 7), and was higher, by 1.72 folds (*p* = 0.0223), with LP10-5X than with vehicle treatment. Trend analysis revealed that LP10 treatment dose-dependently affected the type I muscle fiber. LP10 supplementation could increase type I muscle fiber in gastrocnemius muscle to promote exercise endurance.

## 4. Conclusions

LP10 has anti-fatigue activity by decreasing plasma lactate, ammonia, CK, and serum glucose levels, thereby enhancing exercise performance in mice. In this study, we found that six weeks of supplementation with LPS10 significantly improved the forelimb grip strength and the swimming time to exhaustion of test mice. Exercise-induced fatigue-related parameters including lactate, ammonia, CK, and glucose levels were dose-dependently modulated by LP10 supplementation. LP10 also had beneficial effects on the lipid profile and renal functions. Many studies have demonstrated that *Lactobacillus* spp. has antioxidant activity and immune functions. The possible mechanism by which LP10 increases muscle mass could be that *Lactobacillus* specific strains reduce inflammation, an effect correlated with the improvement of skeletal muscle atrophy markers. *Lactobacilli* counts were reduced in leukemic mice, and this decrease was correlated negatively with the atrophy markers in the muscle. Pro-inflammatory cytokines, such as IL-6 or TNF-α, have been proposed to contribute to muscle atrophy [55]. Concerning lean body mass, in our previous study, we reported that whey protein affected biochemical assessments with long-term aerobic swimming training and enhanced exercise performance without muscle hypertrophy [27]. In this study, we found that LP10 supplementation increased exercise performance, decreased white adipose tissue, increased muscle mass, and enhanced gastrocnemius muscle type I fiber numbers without BW gain. These results suggest that gut microbiota contribute to the host metabolic phenotype to affect physical activity in terms of energy balance and body composition. In fact, the most interesting issue is probiotics combined with exercise performance. Body temperature regulation, reproduction, and tissue growth are energy-dependent processes that may rely in part on gut microbial energy production [13]. We found that LP10 supplementation could enhance glucose utilization to increase endurance exercise time by increasing the number of gastrocnemius type I muscle fibers. We provide evidence that LP10 affects biochemical features with long-term aerobic swimming. For future investigations, LP10 could be used in humans who focus on aerobic endurance training for protective and health purposes. In addition, data in humans supporting the existence of such interplay are lacking. Because humans have different lifestyles, behaviors, diets, and many environmental factors that influence their microbiomes, future studies should be performed in humans to validate the concepts based on animal models.

## Figures and Tables

**Table 1:** General characteristics of mice with *Lactobacillus plantarum* TWK10 (LP10) supplementation.

Characteristic	Vehicle	LP10-1X	LP10-5X	Trend Analysis
Initial BW (g)	29.6 ± 0.2	29.3 ± 0.3	29.2±1.2	0.5370
Final BW (g)	40.1 ± 0.7 ^c^	37.1 ± 0.3 ^a^	38.8 ± 0.1 ^a,b^	0.6493
Food intake (g/day)	6.3 ± 0.1 ^a^	6.2 ± 0.0 ^a^	7.5 ± 0.1 ^b^	<0.0001 (↑)
Water intake (mL/day)	6.9 ± 0.1 ^a^	6.8 ± 0.2 ^a^	7.6 ± 0.0 ^b^	<0.0001 (↑)
*Weight (g)*				
Liver (g)	2.13 ± 0.05	2.10 ± 0.05	2.10 ± 0.03	0.9075
Kidney (g)	0.68 ± 0.02	0.67 ± 0.04	0.72 ± 0.04	0.1272
EFP (g)	0.85 ± 0.07 ^b^	0.55 ± 0.03 ^a^	0.42 ± 0.05 ^a^	<0.0001 (↓)
Heart (g)	0.20 ± 0.01	0.20 ± 0.01	0.20 ± 0.00	0.3908
Lung (g)	0.21 ± 0.01	0.22 ± 0.01	0.21 ± 0.00	0.9353
Muscle (g)	0.36 ± 0.01	0.37 ± 0.01	0.37 ± 0.01	0.4790
BAT (g)	0.13 ± 0.01	0.12 ± 0.00	0.13 ± 0.01	0.9473
*Relative weight (%)*				
Liver	5.29 ± 0.03 ^a^	5.65 ± 0.09 ^b^	5.43 ± 0.06 ^a^	0.1073
Kidney	1.70 ± 0.02 ^a^	1.81 ± 0.02 ^b^	1.86 ± 0.03 ^b^	<0.0001 (↑)
EFP	2.09 ± 0.16 ^c^	1.48 ± 0.09 ^b^	1.08 ± 0.15 ^a^	<0.0001 (↓)
Heart	0.49 ± 0.10 ^a^	0.54 ± 0.07 ^b^	0.53 ± 0.07 ^b^	0.0018 (↑)
Lung	0.53 ± 0.03 ^a^	0.58 ± 0.03 ^b^	0.55 ± 0.01 ^a,b^	0.2009
Muscle	0.90 ± 0.02 ^a^	0.99 ± 0.01 ^b^	0.96 ± 0.02 ^b^	0.0326 (↑)
BAT	0.31 ± 0.01	0.31 ± 0.01	0.33 ± 0.02	0.6881

Data are mean ± SEM, *n* = 8 mice/group. Different letters (a, b, c) in the same row indicate a significant difference at *p* < 0.05. Food efficiency ratio: body weight (BW) gain (g/day)/food intake (g/day). Muscle mass includes both gastrocnemius and soleus muscles in the back part of the lower legs. BAT: brown adipose tissue; EFP: epididymal fat pad. Mice were pretreated with vehicle, LP10-1X, or LP10-5X for six weeks.

**Table 2:** Biochemical analysis with LP10 supplementation at the end of the experiment.

Variable	Vehicle	LP10-1X	LP-5X	Trend Analysis
CK (U/L)	193 ± 36	169 ± 22	181 ± 25	0.8469
TP (g/dL)	4.8 ± 0.1	4.9 ± 0.1	4.9 ± 0.1	0.9571
Albumin (g/dL)	3.6 ± 0.0 ^b^	3.6 ± 0.0 ^b^	3.3 ± 0.1 ^a^	0.012(↓)
BUN (mg/dL)	26.5 ± 0.5 ^b^	22.4 ± 0.6 ^a^	23.0 ± 0.9 ^a^	0.0017(↓)
Creatinine (mg/dL)	0.27 ± 0.01	0.27 ± 0.01	0.29 ± 0.01	0.4627
UA (mg/dL)	0.91 ± 0.03	1.01 ± 0.10	1.03 ± 0.10	0.5858
TC (mg/dL)	143 ± 6	144 ± 6	130 ± 3	0.1804
TG (mg/dL)	205 ± 12 ^b^	159 ± 11 ^a^	151 ± 6 ^a^	0.0005(↓)
Glucose (mg/dL)	166 ± 4	161 ± 5	157 ± 5	0.1336

Data are mean ± SEM, *n* = 8 mice/group. Different letters (a, b) in the same row indicate a significant difference at *p* < 0.05 by one-way ANOVA. CK, creatine kinase; TP, total protein; BUN, blood urea nitrogen; UA, uric acid; TC, total cholesterol; TG, triacylglycerols.
